# Regulation of mitotic clonal expansion and thermogenic pathway are involved in the antiadipogenic effects of cyanidin-3-O-glucoside

**DOI:** 10.3389/fphar.2023.1225586

**Published:** 2023-08-08

**Authors:** Maria Sofia Molonia, Federica Lina Salamone, Claudia Muscarà, Gregorio Costa, Grazia Vento, Antonella Saija, Antonio Speciale, Francesco Cimino

**Affiliations:** ^1^ Department of Chemical Biological Pharmaceutical and Environmental Sciences, University of Messina, Messina, Italy; ^2^ “Prof Antonio Imbesi” Foundation, University of Messina, Messina, Italy; ^3^ Department of Human and Pediatric Pathology “Gaetano Barresi”, University of Messina, Messina, Italy; ^4^ Department of Experimental Medicine (DIMES), University of Genova, Genoa, Italy

**Keywords:** adipogenic process, anthocyanin, cell cycle, differentiation phases, early and late phases, thermogenesis

## Abstract

**Introduction:** Obesity is a metabolic disease with an increase both in cell size (hypertrophy) and in cell number (hyperplasia) following differentiation of new adipocytes. Adipogenesis is a well-orchestrated program in which mitotic clonal expansion (MCE) occurs in the early step followed by the late terminal differentiation one.

**Methods:** Aim of the study was to evaluate the *in vitro* effects of cyanidin-3-O-glucoside (C3G), an anthocyanin present in many fruits and vegetables, in the early or late phase of 3T3-L1 preadipocytes differentiation.

**Results:** C3G exposure in the early phase of adipogenesis process induced a more marked reduction of CCAAT/enhancer-binding protein-β (C/EBPβ), peroxisome proliferator-activated receptor γ (PPAR-ɣ) and fatty acid synthase (Fasn) expression than late phase exposure and these effects were associated to a reduced MCE with cell cycle arrest at G0/G1 phase via p21 expression. Furthermore, C3G exposure during the early phase activated AMP-activated protein kinase (AMPK) pathway better than in the late phase promoting the enhancement of beige-like adipocytes. In fact, C3G induced thermogenic biomarkers uncoupling protein-1 (*Ucp1*) and peroxisome proliferator-activated receptor-gamma coactivator-1 alpha (*Pgc1*) and these effects were more evident during early phase exposure.

**Conclusion:** Our data demonstrate that C3G reduces the terminal adipogenic process affecting the early phase of differentiation and inducing a thermogenic program.

## 1 Introduction

Obesity is among the most important public health issues due to its ever-increasing diffusion in the population. It is a metabolic disorder with increased number (hyperplasia) as well as size (hypertrophy) of adipocytes, with consequent expansion of adipose tissue. Obesity raises the risk of several pathological conditions like insulin resistance, type 2 diabetes mellitus, dyslipidemia, atherosclerosis, and cardiovascular diseases (Rohm et al., 2022). A crucial role in obesity is represented by adipogenesis, the process involving preadipocyte differentiation into mature adipocytes accumulating triglycerides and maintaining energy homeostasis (Saponaro et al., 2015). Adipogenesis is a well-orchestrated program showing mitotic clonal expansion (MCE) in the early step followed by the late terminal differentiation one (Audano et al., 2022). In the early phase, quiescent cells in G0/G1 re-enter S and G2 phases, and cell number increases followed by adipogenic genes expression (Tang et al., 2003a). For this to happen, a downregulation of cyclin-dependent kinase (CDK) inhibitors, like p21, is necessary. Following MCE, cells undergo differentiation expressing and activating adipogenic transcription factors, like CCAAT/enhancer-binding proteins (C/EBPs) and peroxisome proliferator-activated receptor γ (PPAR-γ) (Mota de Sá et al., 2017). C/EBPβ and C/EBPδ rise in the early phase following hormonal stimulation (Hishida et al., 2009) and induce PPAR-γ and C/EBPα, which control terminal differentiation (Guo et al., 2015). Additionally, PPAR-γ induces a set of responses involving the expression of the key fatty acid synthesis-related genes such as fatty acid synthase (*Fasn*), acetyl-CoA carboxylase (*Acc1*), and sterol regulatory element-binding protein-1 (S*rebp*1).

It is acknowledged that obesity results from an imbalance between energy expenditure and consumption so inducing surplus energy storage as lipids in white adipose tissue (WAT) (Crane et al., 2015). However, in particular conditions, WAT can show brown fat–like phenotype (beige adipocytes) that can dissipate energy as heat with an increased expression of thermogenesis-associated genes. AMP-activated protein kinase (AMPK) activation in involved in beige adipocyte differentiation and function since it inhibits fatty acids synthesis and promotes their oxidation, and it regulates mitochondria biogenesis by phosphorylating the peroxisome proliferator-activated receptor-gamma coactivator-1 alpha (*Pgc1*) that directs the expression of thermogenic genes, of which uncoupling protein-1 (*Ucp1*) is of central importance (Cantó et al., 2009). Additionally, studies demonstrated that AMPK also inhibits MCE together with a decreased expression of early and late adipogenic factors so affecting adipogenesis process (Habinowski and Witters, 2001; Bijland et al., 2013). All these activities are controlled by G protein-coupled receptors through downstream signals involving G protein complexes. In particular, beta adrenoceptors induce lipolysis and thermogenesis via AMPK in adipocytes (Im et al., 2021). Therefore, targeting the molecular mechanisms involved in differentiation of adipocyte precursor cells can be considered as a suitable therapeutic approach against obesity and related disorders (Haider and Larose, 2019; Ambele et al., 2020).

Many dietary natural compounds have been shown to prevent obesity by modulating preadipocyte proliferation and/or differentiation, apoptosis of adipocytes, lipid metabolism, insulin sensitivity, and the activation of thermogenic program (Bonet et al., 2013). Among these, anthocyanins showed *in vitro* and *in vivo* anti-obese property preventing and managing the over-weight condition and related comorbidities (Sivamaruthi et al., 2020). In particular, several studies reported the ability of the anthocyanin cyanidin-3-O-glucoside (C3G) to improve adipose tissue dysfunction, restoring insulin signaling and reducing inflammation in hypertrophic 3T3-L1 murine adipocytes (Molonia et al., 2020; Molonia et al., 2021b). However, despite the several data reported in the literature, few studies have investigated the molecular mechanisms underlying C3G effects on the main steps of the adipogenesis process (Matsukawa et al., 2017). For this reason, we studied the effects of C3G on adipogenesis and thermogenesis by exposing 3T3-L1 preadipocytes to this anthocyanin during the early or the late phase of differentiation.

## 2 Materials and methods

### 2.1 Materials

The anthocyanin C3G, in a high HPLC purity grade (>97%) was purchased from Polyphenols AS (Sandnes, Norway). Dimethyl sulfoxide (DMSO) and isopropanol, in their highest commercially available purity grade, were provided from Carlo Erba Reagent (Milan, Italy). All other reagents, unless explicitly stated otherwise, were supplied from Sigma Aldrich (Milan, Italy).

### 2.2 Cell cultures and treatments

Murine 3T3-L1 pre-adipocytes (American Tissue Culture Collection, Manassas, VA, United States; ATCC CL-173; RRID:CVCL_0123), were cultured in growth medium (DMEM with 10% NBCS, 100 U/mL penicillin/streptomycin solution, 4 mM L-glutamine, 25-mM HEPES buffer) at 37°C in a humidified chamber with 5% CO_2_. Cells were subcultured every 4 days at approximately 90% confluence.

**FIGURE 1:**
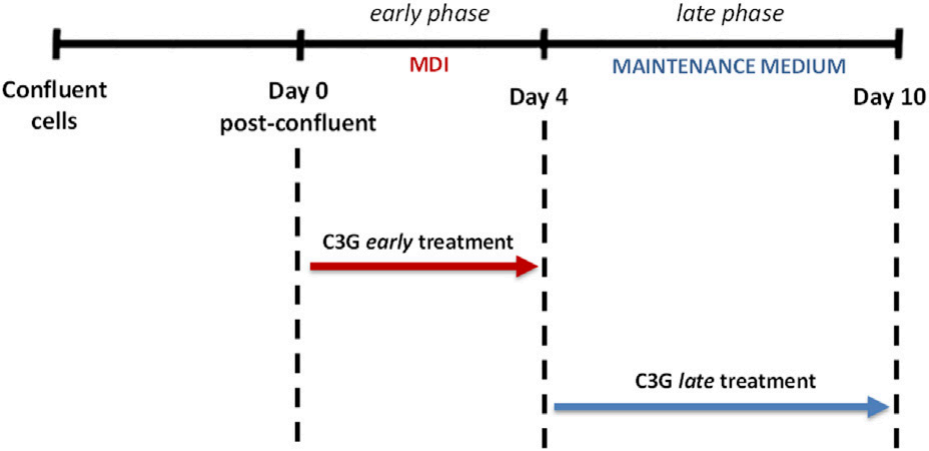
Schematic representation of the two different C3G (5–10 μM) exposure periods (early or late) during adipocyte differentiation.

To obtain totally differentiated cells, 3T3-L1 were seeded at 1.3 × 10^4^ cells/cm^2^ in multiwell plates and maintained in culture for 10 days after the confluence. In detail, the cells were incubated in the first 4 days (early phase) with DMEM with 10% FBS, 4 mM L-glutamine, 100 U/mL penicillin/streptomycin solution and 25-mM HEPES buffer, containing differentiation-inducing agents (0.5 mM IBMX, 1 μΜ DEX and 1 μg/mL insulin [MDI]). Subsequently the cells were maintained, for the following 6 days (late phase), in DMEM with 10% FBS (see above), [Maintenance Medium], until total differentiation in mature adipocytes (Figure 1).

In this last phase the medium was refreshed every 3 days: from day 4 to day 7 the cells were culture with a medium containing 1 μg/mL insulin, while from day 7 to day 10 the cells were incubated in an insulin-free DMEM.

For the experiments, cells were incubated with C3G at two concentrations (5 and 10 μM) during the differentiation process. These concentrations, considered not cytotoxic (Supplementary Figure S1), are chosen since within a cumulative circulating range of parent drugs and their metabolites, physiologically reachable following the consumption of anthocyanins (Czank et al., 2013; de Ferrars et al., 2014a; de Ferrars et al., 2014b; Speciale et al., 2014). As shown in Figure 1, C3G has been added in the early phase or in the late phase, for examining its activity on the two phases of adipogenic differentiation.

In order to evaluate the involvement of AMPK in C3G antiadipogenic effect, adipocytes were treated with the AMPK inhibitor Compound C (CC, 100 nM) in presence or not of C3G (5 and 10 μM).

For the analysis of mitotic clonal expansion process and for the AMPK inhibition studies, the cells were processed at the end of the early phase (after 4 days); all the other endpoints were evaluated once differentiation process was completed (after 10 days). In all experiments C3G was freshly solubilized in DMSO and instantly used. The DMSO concentration in the culture medium during the treatments was always ≤0.025% (v/v). Cells treated with the vehicle alone (DMSO 0.025% v/v) and later exposed in the early phase to the MDI differentiation inducers and in the late phase to Maintenance medium were used as controls (CTR).

### 2.3 Oil Red O assay

To evaluate C3G effect on lipid accumulation, Oil Red O staining was carried out after the differentiation process was completed (day 10) following the methodology reported in our previous paper (Muscarà et al., 2019). After staining, the cultures were observed by optical microscope and photographed (×40 magnification). Subsequently the Oil Red O stain kept within the cells was eluted with 100% isopropanol and lipid accumulation was determinated by spectrophotometric analysis at 490 nm using a microplate reader (GloMax^®^ Discover System-TM397, Promega Corporation, WI, United States). Results were expressed as fold change against control.

### 2.4 Cell lysates preparation

For total lysate preparation 3T3-L1 cells were treated with a homogenizer lysis buffer (1 mM EDTANa_2,_ 10 mM Tris–HCl, 150 mM NaCl, 1% Tryton, 0.1% SDS and 5% glycerol) containing protease inhibitors (5 mM NaF, 2 μg/mL aprotinine, 1 mM benzamidine and 1 μg/mL leupeptin) and 1 mM dithiothreitol (DTT). In addition, to further promote cell lysis, the pellet was passed five times in a thin needle and maintained on ice in agitation for 60 min. Afterwards, the samples were centrifuged at 15,000 g for 15 min at 4°C and the supernatant with the proteins was collected and maintained at −20°C until use.

The protein concentration in lysates was then determined by the Bradford assay (Bradford, 1976).

### 2.5 Western blot analysis

For immunoblot analyses, 30 μg of denatured proteins were subjected to SDS-PAGE on 10% acrylamide/bisacrylamide gels. Then, the separated proteins were transferred to a Polyvinylidene Difluoride (PVDF) membranes (Hybond-P PVDF, Amersham Bioscience, Milan, Italy), and, after incubation with a blocking solution (5% of non fat dried milk), the membranes were incubated overnight at 4°C with the following specific primary antibodies diluted in TBS-T buffer (10 mM Tris-base, 100 mM NaCl and 0.1% Tween 20): mouse anti-PPAR-γ monoclonal antibody (#sc-7273 Santa Cruz Biotechnology, Dallas, TX, United States) (1:1,500), rabbit anti-pAMPK (Thr172) monoclonal antibody (Cat# 2535 Cell Signaling Technology, Danvers, MA, United States; RRID:AB_331250) (1:1,000), rabbit anti C/EBPβ (LAP) polyclonal antibody (Cat# 3087 Cell Signaling Technology, Danvers, MA, United States; RRID:AB_2078052) (1:1,000), rabbit anti p21 Waf1/Cip1 monoclonal antibody (Cat# sc-271610 Santa Cruz Biotechnology, Dallas, TX, United States; RRID:AB_10647231) (1:500), rabbit anti-β-Actin monoclonal antibody (Cat# 4970 Cell Signaling Technology, Danvers, MA, United States; RRID:AB_2223172) (1:6,000). Then the membranes were subjected to 2 h incubation at room temperature with peroxidase-conjugated (HRP) secondary antibody labeled goat antirabbit Ig (Cat# 7074 Cell Signaling Technology, Danvers, MA, United States; RRID:AB_2099233) (1:6,000) or goat anti-Mouse IgM Secondary Antibody, HRP conjugate (Cat# 7076 Cell Signaling Technology, Danvers, MA, United States; RRID:AB_330924) (1:6,000), and developed using an ECL plus detection reagent (Amersham Biosciences, Milan, Italy). Quantitative analysis was achieved by densitometry using ImageJ software (v1.53; Wayne Rasband, National Institutes of Health in Bethesda, Maryland, United States; RRID:SCR_003070).

### 2.6 Real time PCR

Total RNA was extracted using E. Z.N.A.^®^ Total RNA kit (OMEGA Bio-Tek, VWR, Milan, Italy), quantified by Quanti-iT TM RNA assay kit QUBIT (Invitrogen, Milan, Italy), and reverse transcripted with M-MLV Reverse Transcriptase. Quantitative real-time polymerase chain reaction (PCR; Applied Biosystems 7300 Real-Time PCR System, CA, United States) coupled with SYBR green chemistry (SYBR green JumpStart Taq Ready Mix) was carried out for identification of mRNA levels of *Fabp4* (FW 5′-AAG​GTG​AAG​AGC​ATC​ATA​ACC​CT-3′, RV 5′-TCA​CGC​CTT​TCA​TAA​CAC​ATT​CC-3′) (Molonia et al., 2022), *Fasn* (FW 5′-GGA​GGT​GGT​GAT​AGC​CGG​TAT-3’, RV 5’-TGG​GTA​ATC​CAT​AGA​GCC​CAG-3’) (Zhang et al., 2016)*, Acc1* (FW 5’-GAG AGG GGT CAA GTC CTT CC -3', RV 5'- CTG CTG CCG TCA TAA GAC AA-3') (Soundharrajan et al., 2020), *Srebp1* (FW 5’-TGG CTT GGT GAT GCT ATG TT-3', RV 5’- TAA GGG GTT GGG AGT AGA GG-3’) (Ning et al., 2017), *Ucp1* (FW 5′-ACT​GCC​ACA​CCT​CCA​GTC​ATT-3’, RV 5’-CTT​TGC​CTC​ACT​CAG​GAT​TGG-3’) (Asano et al., 2014)*, Pgc1* (FW 5′-TGT​GGA​ACT​CTC​TGG​AAC​TGC-3’, RV 5’-GCC​TTG​AAA​GGG​TTA​TCT​TGG-3’) (Asano et al., 2014)*.* 18S rRNA (FW 5′-GTA​ACC​CGT​TGA​ACC​CCA​TT-3′, RV 5′-CCA​TCC​AAT​CGG​TAG​TAG​CG-3′) (Ferrari et al., 2017) was chosen as housekeeping gene as our preliminary investigations indicated its expression did not change during differentiation. Data were analyzed with the SDS 1.3.1 software (Applied Biosystems, Foster City, CA, United States) and expressed as threshold cycle (Ct). The fold increase of mRNA expression compared with the control cells was calculated using the 2^−ΔΔCT^ method (Livak and Schmittgen, 2001).

### 2.7 Cell count analysis

Cell count was examined on day 4 of differentiation (early stage) by Erythrosin B exclusion test. Erythrosin B was prepared as a 0.1% solution in DPBS according to the manufacturer’s instructions (Sigma Aldrich). After trypsinization, cell number was determined by hemocytometer counts and expressed as percentage vs. MDI differentiated control.

### 2.8 Cell cycle analysis

Postconfluent 3T3-L1 cells were differentiated with the addition of MDI in the presence or absence of C3G for 4 days (early stage) and cell cycle distribution was determined by DNA staining with BD Cycletest™ Plus DNA Kit according to Allegra et al., (2018).

### 2.9 Statistical analysis

All the experiments were carried out on four experimental units (*n* = 4) each performed in triplicate. The choice of sample size is based on prior experience (Molonia et al., 2021a; Molonia et al., 2021b; Molonia et al., 2022). For all assays technical triplicates were used to ensure the reliability of single values. Results are expressed as mean ± SD from three experiments and subjected to a one-way ANOVA test, followed by Tukey’s HSD, ezANOVA software (http://www.sph.sc.edu/comd/rorden/ezanova/home.html). Differences were considered significant for *p* < 0.05.

## 3 Results

### 3.1 C3G reduces lipid accumulation in mature adipocytes

**FIGURE 2:**
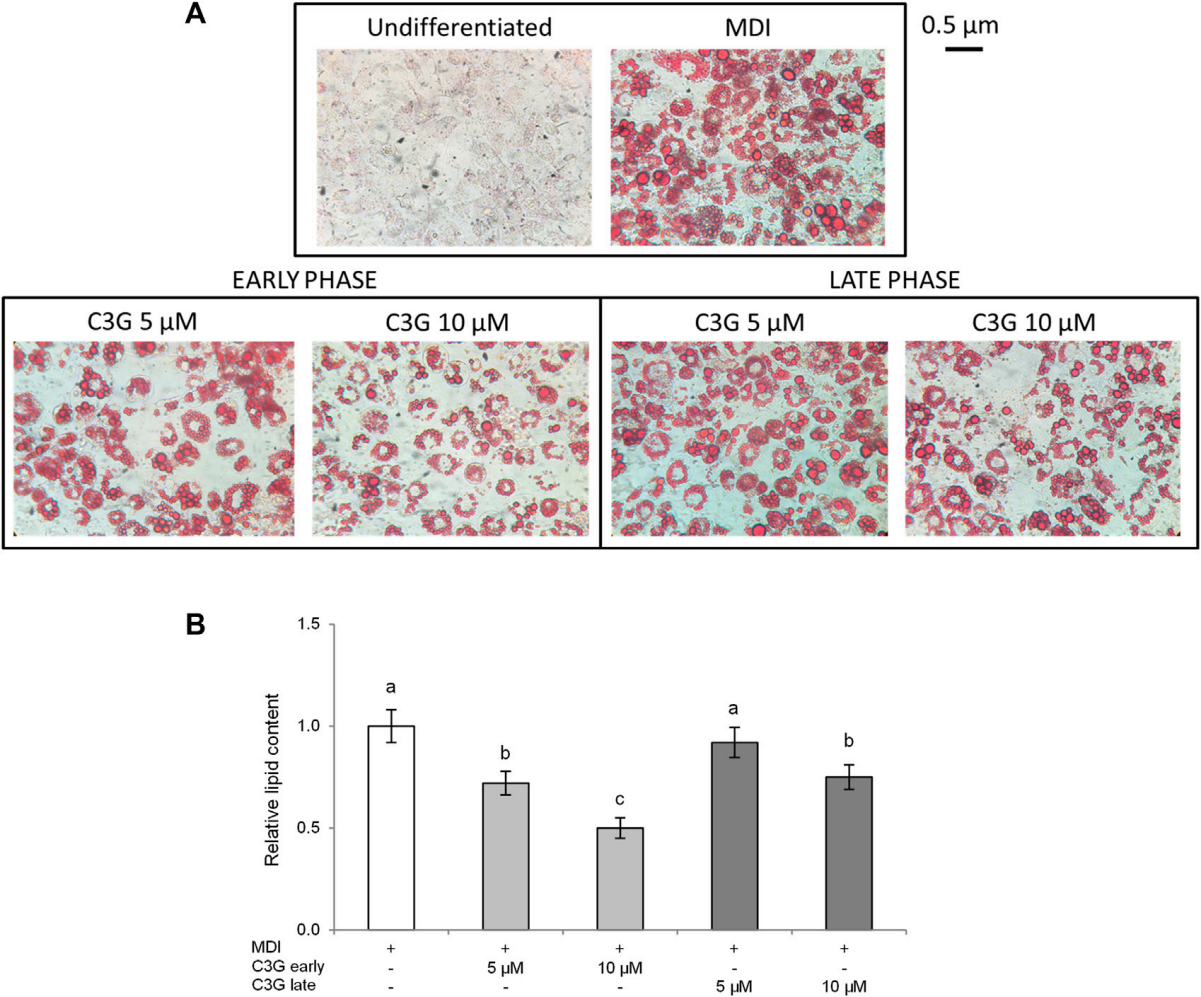
C3G effect on lipid accumulation in 3T3-L1 cells treated in early or late phase. **(A)** Staining with Oil Red O (magnification at × 40). Representative images of four separate experiments. **(B)** Lipids were extracted by using isopropanol and expressed as relative content vs. CTR cells. All data are expressed as mean ± SD of four independent experiments (*n* = 4) each in triplicate. Means with the same letter are not significantly different from each other (*p* > 0.05).

In order to evaluate the protective effect of C3G in the different stages of the adipogenesis process, we estimated the activity of this anthocyanin on intracellular lipid accumulation using the histological technique of staining with Oil Red O. In particular, C3G has been added to 3T3-L1 preadipocytes during the early (day 0 to day 4) or the late phase (day 4 to day 10). A noticeable increase in the number and size of lipid deposits can be observed in CTR cells (MDI differentiated, see Materials and Methods Section 2.2 for details) with respect to undifferentiated cells (Figure 2A). Conversely, C3G treatment, at both tested concentrations (5–10 µM), reduced the amount of lipid deposits. These effects were more evident in cells exposed to C3G during the early phase compared to the late phase (Figure 2B). Therefore, the addition of C3G in the induction phase of the 3T3-L1 differentiation process resulted in a marked suppressive activity of adipogenesis.

### 3.2 C3G showed anti-adipogenic effects during adipocyte differentiation

It is widely known that the adipogenesis process is activated and regulated by several signaling cascades of transcription factors and enzymes (Jakab et al., 2021). Thus, to assess the mechanism through which C3G can prevent accumulation of intracellular lipids especially in the *early phase* of differentiation, expression of C/EBPβ and PPAR-γ was evaluated. C/EBPβ is a crucial transcription factor expressed in the early phase of adipocyte differentiation and stimulated promptly following adipogenic stimuli and accountable for PPAR-γ activation (Guo et al., 2015). It has also been shown that reduced levels of C/EBPβ inhibit C/EBPα and PPAR-γ gene expression, suppressing adipogenesis so supporting the function of C/EBPβ in terminal adipogenic differentiation (Tanaka et al., 1997).

**FIGURE 3:**
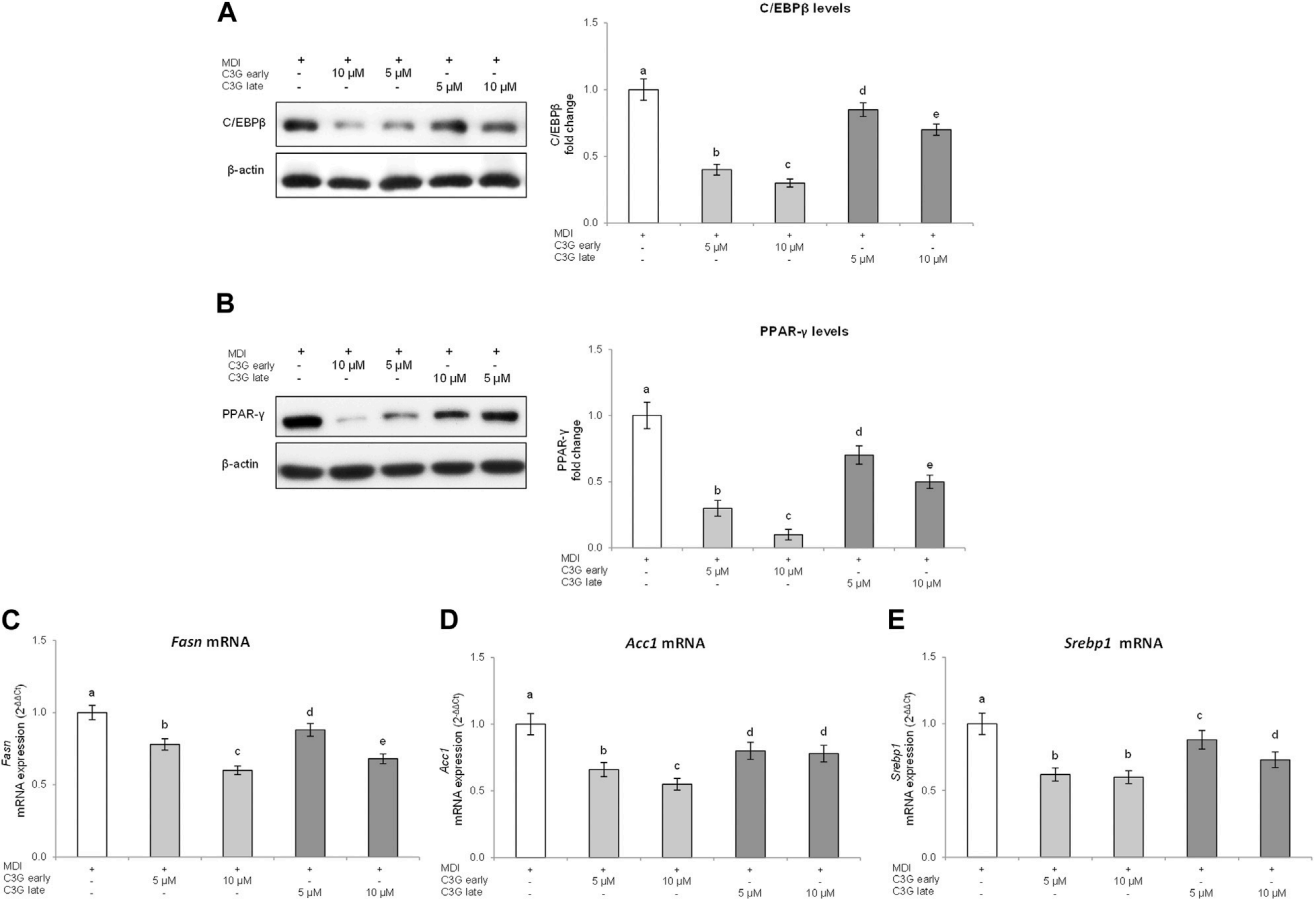
C3G effects on C/EBPβ **(A)** and PPAR-γ **(B)** protein levels, and on *Fasn* **(C)**, *Acc1* **(D)** and *Srebp1* **(E)** gene expression in 3T3-L1 cells treated in early or late phase. **(A,B)** Representative images from four independent experiments. Densitometry data are expressed as fold change vs. MDI control. The order of histogram bars was rearranged for illustrative purposes. **(C)** *Fasn*, *Acc1* and *Srebp1* mRNA expression levels were analyzed by real time PCR. All data are expressed as mean ± SD of four independent experiments (*n* = 4) each performed in triplicate. Means with the same letter are not significantly different from each other (*p* > 0.05).

The results obtained (Figure 3A) show how C/EBPβ expression was significantly decreased following C3G treatment compared to MDI control cells. Interestingly, C3G was more effective during early phase exposure compared to the late one. Therefore, given the significance of C/EBPβ in adipocyte differentiation, inhibition of its expression and nuclear translocation by C3G during early phase, may be enough to stop terminal differentiation.

It is widely demonstrated how, in the adipogenesis process, C/EBPs work sequentially and cooperatively with PPARs proteins family, in order to induce terminal differentiation (Ali et al., 2013). PPAR-γ is a member of PPAR family of nuclear hormone receptors, considered the main modulator of adipogenesis process (Lefterova et al., 2014) since it increases intracellular lipids by acting on lipid metabolism and regulates energy homeostasis being able to modulate the expression of several transcription factors and proteins, like C/EBPα and the sterol regulatory element binding protein-1 (SREBP-1) (Rodriguez-Cuenca et al., 2012). Thus, to explore in more detail, the mechanisms by which C3G exerts its effects we evaluated the PPAR-γ levels. C3G treatment, added in the different stages of the differentiation process, reduced PPAR-γ compared to MDI control cells (Figure 3B). Also in this case it is possible to observe that C3G exposure in the early phase of adipogenesis induced a more marked reduction of PPAR-ɣ. These results confirm data obtained with Oil Red O. As a further demonstration of the antiadipogenic effect of C3G treatment in 3T3L1 cells, the gene expression of lipogenic genes such as *Fasn*, *Acc1* and *Srebp1* was then evaluated.

The data obtained confirmed the same trend observed for PPAR-ɣ. C3G treatment, particularly during the early phase, significantly inhibited *Fasn*, *Acc1* and *Srebp1* mRNA expression compared to MDI control cells (Figure 3C). These findings thereby suggest and confirm that inhibition of adipocyte maturation by C3G is correlated with the inhibition of the expression of adipogenic transcription factors especially throughout the early phase of adipocyte differentiation.

### 3.3 C3G affects mitotic clonal expansion process

As mentioned above, adipogenesis is a complicated process involving modifications in cell morphology, gene expression, and sensitivity to hormonal alterations (Cristancho and Lazar, 2011). In particular, in the initial stage of the adipogenic differentiation process, when stimulated to differentiate by hormones, growth-arrested 3T3-L1 preadipocytes simultaneously return into cell cycle and undertake cell proliferation so that begin MCE, which is needed for the adipocyte terminal differentiation (Lazar et al., 2018). Since this event takes place at the early stage of adipocyte differentiation, to better elucidate C3G effects on MCE, we performed a flow cytometry analysis on the fourth day, namely, at the end of induction step.

**FIGURE 4:**
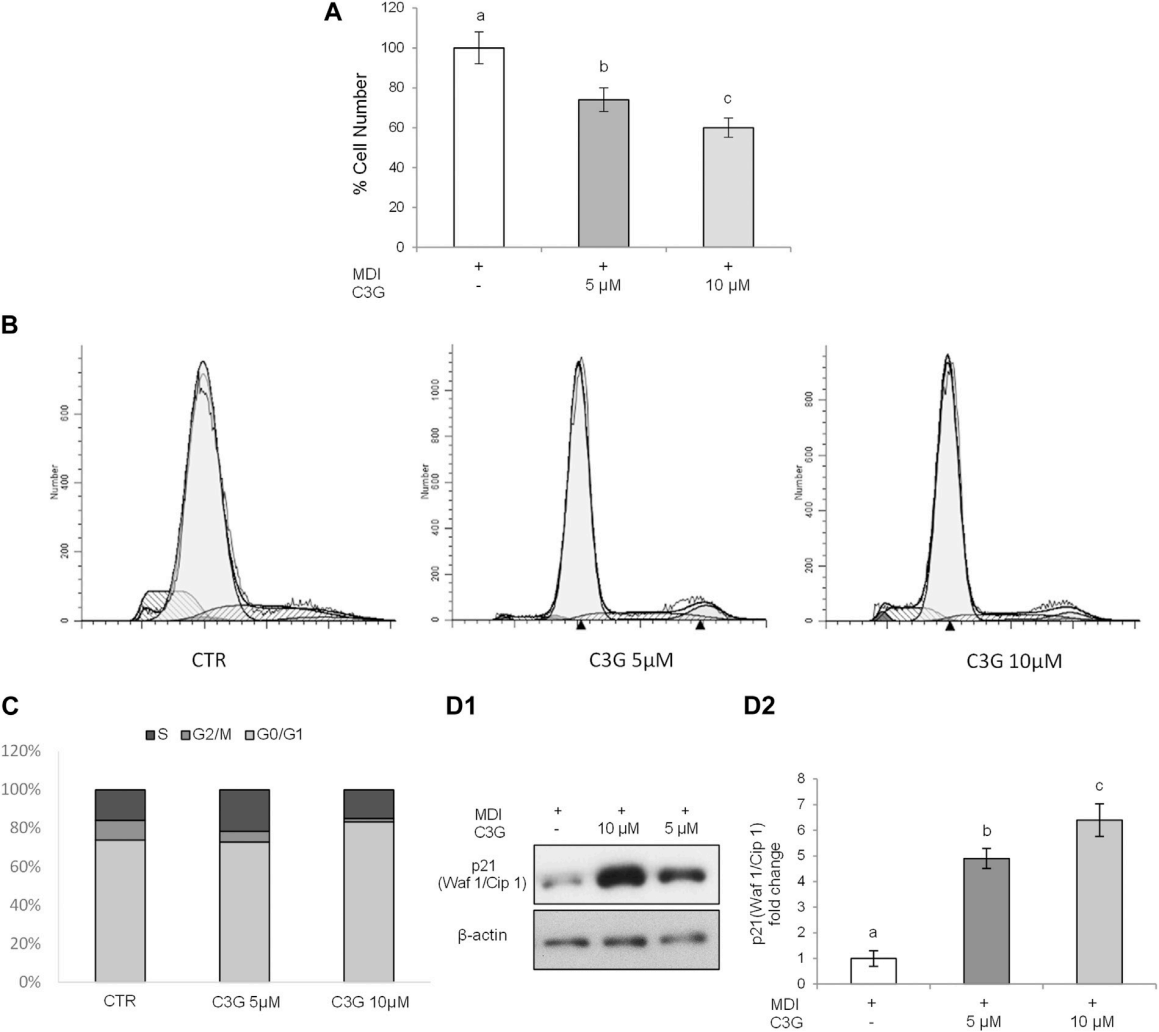
C3G effect on Mitotic Clonal Expansion in 3T3-L1 cells treated in early phase. **(A)** Effect on cell number: cell count was determined after trypsinization on day 4 by hemocytometer counts. **(B–C)** Effect on cell cycle arrest. **(D)** Effect on p21 protein levels*:* **(D1)** Representative images from four independent experiments. **(D2)** Densitometry data are reported as fold change vs. MDI control. The order of histogram bars was rearranged for illustrative purposes. All data are expressed as mean ± SD of four independent experiments (*n* = 4) each in triplicate. Means with the same letter are not significantly different from each other (*p* > 0.05).

The results obtained show that the C3G exposure through the early phase of differentiation, determined a cell number reduction compared to MDI control cells, supporting that C3G inhibited the re-entry of preadipocytes in the cell cycle in the MCE stage (Figure 4A).

We further investigated the cell cycle progression of adipocytes during MCE. The results reported in Figures 4B, C show cell cycle distribution profile of 3T3-L1 cells following C3G treatment in early phase. In particular, it is possible to observe how, respect to MDI control cells, C3G 5µM and 10 µM caused a clear decrease of G2/M phases (10.3% for MDI and 6.9% and 1.8% for C3G 5 and 10 µM respectively) and an arrest of the cell cycle at the G0/G1 phase at the higher C3G concentration tested (73.7% for MDI and 83.5% for C3G 10 µM) (Figure 4C).

Since the cell cycle progression in mammals is regulated by several complexes of cyclins and cyclin-dependent kinases, in order to better understand the C3G mechanism, we consequently evaluated the levels of p21, a well-known CDK inhibitor, whose sustained expression promotes cell cycle arrest at G1 phase (Tang and Lane, 2012). The results obtained clearly show how, even in this case, C3G treatment induced the expression of p21 respect to MDI control cells (Figure 4D). Altogether, thereby, these data suggest that the antiadipogenic effect of C3G in the early phase is due to the ability of this anthocyanin to inhibit clonal expansion during adipogenic differentiation through the induction of G0/G1 cell cycle arrest by modifying the expression of p21.

### 3.4 C3G protective effects involves the AMPK pathway

Adipose tissue is an energetic storage organ where adipocytes have an important role in preserving lipid homeostasis and energy balance through storage of triacylglycerols or release of free fatty acids depending on modifications of energy needs (Ali et al., 2013).

In this regard, since AMPK stimulates the development of beige-like phenotype from white adipose tissue inducing a thermogenic program, special attention has been given to this signaling pathway. In fact, phosphorylate, AMPK induces catabolic pathways, as fatty acid oxidation, and inhibits energy-consuming pathways, as fatty acid synthesis (Feng et al., 2016). Furthermore, as adipogenesis is energy-consuming, AMPK negatively regulates adipogenesis also affecting adipocytes differentiation via suppression of the MCE phase, with lower expression of early and late adipogenic factors (Bijland et al., 2013; Kang et al., 2013; Jang et al., 2015).

**FIGURE 5:**
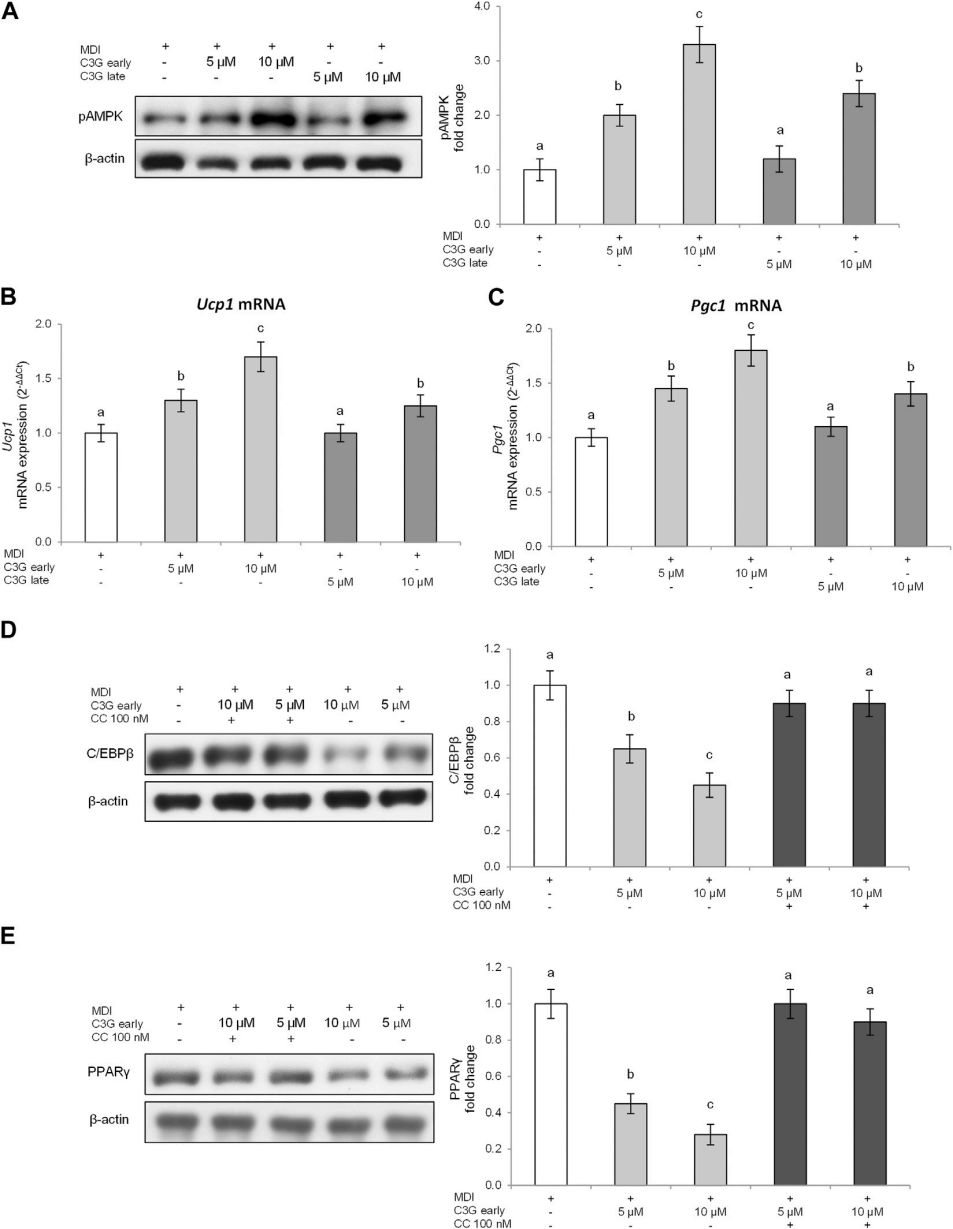
C3G effect on pAMPK levels **(A)**, *Ucp1* **(B)** and *Pgc1* **(C)** gene expression in 3T3-L1 cells treated in early or late phase, and expression of CEBP/β **(D)** and PPARγ **(E)** with the AMPK inhibitor (Compound C—100 nM) in 3T3-L1 cells treated during the early phase. Densitometry data are reported as fold change vs. MDI control. *Ucp1* **(B)** and *Pgc1* **(C)** mRNA expression levels were analyzed by real time PCR. All data are expressed as mean ± SD of four independent experiments (n = 4) each performed in triplicate. Means with the same letter are not significantly different from each other (*p* > 0.05).

Results reported in Figure 5A demonstrate that, in our experimental conditions, C3G treatment during the early phase of the adipogenesis process significantly increases AMPK levels, at both tested concentrations (5–10 μM), respect to control cells. Treatment during the late phase resulted, instead, in a modest phosphorylation of AMPK and exclusively with the greatest dose of C3G (10 μM).

Subsequently, in order to confirm and further explore in more detail the molecular mechanisms by which this anthocyanin activates the thermogenesis process, gene expression levels of *Ucp1* and *Pgc1*, two of the main markers implicated, was evaluated. *Ucp1* is, in fact, the main responsible for the activation of thermogenesis and browning in white adipose tissue, and thus for the production of heat via respiratory uncoupling reactions (Shan et al., 2016). *Pgc1*, on the other hand, in addition to being the main regulator of Ucp1-mediated thermogenesis, is also involved in several cellular mechanisms like glucose and fatty acid metabolism, mitochondrial biogenesis and oxidative metabolism, with consequent protective effects against obesity and related disorders (Uldry et al., 2006).

The obtained data confirm that C3G treatment during the early phase*,* at both the tested concentrations (5–10 μM), significantly increased *Ucp1* (Figure 5B) and *Pgc1* (Figure 5C) gene expression compared to MDI control cells. However, even in this case, the activation of these markers is more marked in the early phase treatment, thus confirming how C3G activates AMPK pathway better during the initial stage of adipocyte differentiation.

Additionally, in order to evaluate the role of AMPK in the protective effects of C3G, we used an AMPK inhibitor (Compound C [CC]) during C3G early exposure and the main adipogenesis markers were determined. Our data confirmed that CC inhibited C3G effects on C/EBPβ (Figure 5D) and PPAR-γ (Figure 5E) in the early phase restoring their levels to MDI control and thus confirming that AMPK activation plays an important role in the protective effects observed.

## 4 Discussion

All the data obtained demonstrated how the C3G treatment decreased accumulation of intracellular lipids in 3T3-L1 cells, and these effects were associated with the inhibition of PPAR-ɣ pathway and C/EBPβ expression, two key transcriptional regulators of the adipogenesis process. The effects on PPAR-ɣ pathway inhibition were also previously demonstrated using an anthocyanin rich extract in hypertrophic 3T3-L1 adipocytes (Muscarà et al., 2019). Interestingly, our data showed that C3G exposure in the early stage of the adipogenic process determined a marked reduction of these transcription factors, compared to the treatment in the late phase*.* At the same extent, early phase C3G treatment reduced intracellular lipids better than late phase, clearly indicating that C3G is able to affect the adipogenesis process during the induction period of the differentiation of preadipocytes. Many dietary bioactive phytochemicals have been previously reported for their effects on adipocyte differentiation above all in the early phase of adipogenesis, in fact, this first stage represent a potential target for obesity prevention (Chang and Kim, 2019). During this stage, phytochemicals can affect cellular proliferation by cell-cycle arrest and related cell signaling pathways involved in the aforesaid processes.

To elucidate the molecular mechanisms underlying C3G-induced anti-adipogenic effect, especially in the first stages of the adipogenic process, we measured C3G effects on MCE. This phenomenon, in fact, occurs early in adipogenic differentiation and is essential for subsequent final differentiation (Tang et al., 2003b). Throughout this phase preadipocytes carry out one or two replications, with reduced CDK inhibitory proteins p21 and p27. Our results demonstrated that C3G significantly suppressed MCE in the early stage, causing block of preadipocytes proliferation of preadipocytes and inducing cell-cycle-arrest in the G1 phase. These effects were associated with activation of CDK inhibitory protein p21.

From the results obtained, we can thereby speculate that the antiadipogenic effect of C3G during the first step of the differentiation process has to be associated with MCE process inhibition, so inhibiting the final differentiation in 3T3-L1 adipocytes. These data are in agreement with recent reports demonstrating how the inhibition of adipogenesis process is directly linked to the modulation of MCE through cell cycle arrest induced by CDK inhibitory protein (Kwon et al., 2012; Abood et al., 2018; Chang and Kim, 2019; Oh and Chun, 2022).

Several evidence has shown that AMPK pathway is involved in modulation of clonal expansion phase and thus in adipocyte differentiation (Habinowski and Witters, 2001). In this context AMPK, a central regulator of energy metabolism in adipose tissue, determines the phosphorylation of several target genes that control cell growth and differentiation of adipose tissue (Day et al., 2017). Furthermore, it was demonstrated that AMPK is closely related to the activation of the thermogenic process regulating mitochondria biogenesis with a consequent raise in the expression of a brown-like phenotype involving thermogenic genes (including *Pgc1*, *Ucp1* and *Prdm1*6) (Yang et al., 2016). In fact, *Ucp1* and *Pgc1* genes are directly regulated by this pathway and AMPK inhibition attenuates the browning of 3T3-L1 adipocytes (Kim et al., 2017; Zheng et al., 2023). Since adipogenesis is an energy-consuming process, AMPK works as a negative regulator of adipogenesis and thus could be an important target in obesity.

With the purpose of investigating further the molecular mechanism behind the antiadipogenic effect of C3G during early or late phases of adipocytes differentiation, the activation of AMPK was examined. Our data have shown how the treatment with C3G resulted in increased levels of AMPK phosphorylation. Also in this case these effects were more pronounced in the first step of the adipogenesis process (Figure 5A). Our data are in accordance with those earlier described by Guo and associates evidencing the *in vitro* effects of C3G on AMPK pathway activation in 3T3-L1 adipocytes and HepG2 hepatoma cells (Guo et al., 2012a; Guo et al., 2012b). In our experiments, the phosphorylation of AMPK was also connected with an increase of the expression of two of the most important genes involved in the thermogenic process *Ucp1* and *Pgc1* (Figures 5B, C). Additionally, using an AMPK inhibitor, we confirmed that the C3G-mediated inhibition of adipogenesis in early phase is directly related with the activation of AMPK pathway.

Taken together these data therefore suggest how C3G, through molecular events that occur mainly in the first stage of the adipocyte differentiation, leads to inhibition of adipogenesis probably via inhibition of MCE promoting the maintenance of the undifferentiated state, and induction of a beige-like phenotype. Therefore, C3G, inhibiting early adipogenic markers and enhancing transcription inhibitors associated to adipogenesis, blocks the final adipogenic differentiation.

These data thereby, help to clarify the molecular basis for the inhibitory mechanism of C3G against adipogenesis *in vitro*, so supporting additional *in vivo* research to validate the role of this anthocyanin in all the clinical conditions characterized by increased adipocytes differentiation and fat accumulation. However, anthocyanins main limitation in clinical usage is represented by their low bioavailability, although new nanotechnology approaches were developed to improve their bioaccessibility and bioavailability (Salehi et al., 2020).

## Data Availability

The raw data supporting the conclusion of this article will be made available by the authors, without undue reservation.
