# Supplementary material for: Regulation of mitotic clonal expansion and thermogenic pathway are involved in the antiadipogenic effects of cyanidin-3-O-glucoside
Source: Front Pharmacol. 2023 Aug 8;14:1225586. doi: 10.3389/fphar.2023.1225586 (PMC10442822; doi:10.3389/fphar.2023.1225586)
Supplement: Supplementary file 1 [file Table1.DOCX]

Supplementary Material

Regulation of mitotic clonal expansion and thermogenic pathway are involved in the antiadipogenic effects of cyanidin-3-O-glucoside

Maria Sofia Molonia^1,2^, Federica Lina Salamone^1*^, Claudia Muscarà^1^, Gregorio Costa^3^, Grazia Vento^4^, Antonella Saija^1^, Antonio Speciale^1†^, Francesco Cimino^1†^

*** Correspondence:** Federica Lina Salamone: federica.salamone@studenti.unime.it

# C3G in vitro citotoxicity

## Method

The cytotoxic effects of C3G (10-250 µM) in 3T3-L1 preadipocytes was evaluated by sulforhodamine B assay (Anwar et al., 2016).

In detail, cells were plated at 2.6 x 10^4^ cells/cm^2^ in multiwell plates and were cultured in growth medium (DMEM supplemented with 10% NBCS, 100 U/ml penicillin/streptomycin solution, 4 mM L-glutamine, 25‐mM HEPES buffer) at 37℃ in a humidified atmosphere of 5% CO_2_. After 24 h semi-confluent monolayers were treated with different concentrations of C3G (10-250 µM in DMSO [0,1% v/v]) for 48h. Control cells were treated with the vehicle (DMSO 0,1% v/v) alone. At the end of time exposure, the cells were fixed using 10% trichloroacetic acid (w/v) for 1 h at 4°C and then washed twice with water and incubated with sulforhodamine B (0.4% w/v in 1% acetic acid) for 30 min at RT, followed by four washes with 1% acetic acid. The dye trapped in the cells was dissolved in 10 mM Tris base solution and the absorbance was measured at 565 nm using a microplate reader (GloMax® Discover System-TM397). Cell viability results are reported as percentage of viable cells with respect to untreated cells.

## Results

The obtained data showed that following the 48h treatment, C3G had no significant effect on 3T3-L1 cell viability at concentrations ≤ 100 𝜇M (Supplementary Figure 1). Therefore, the concentrations used in the present study do not affect, in any way, cell viability.

# Supplementary Figures and Tables

For more information on Supplementary Material and for details on the different file types accepted, please see [here](https://www.frontiersin.org/guidelines/author-guidelines#supplementary-material).

## Supplementary Figures


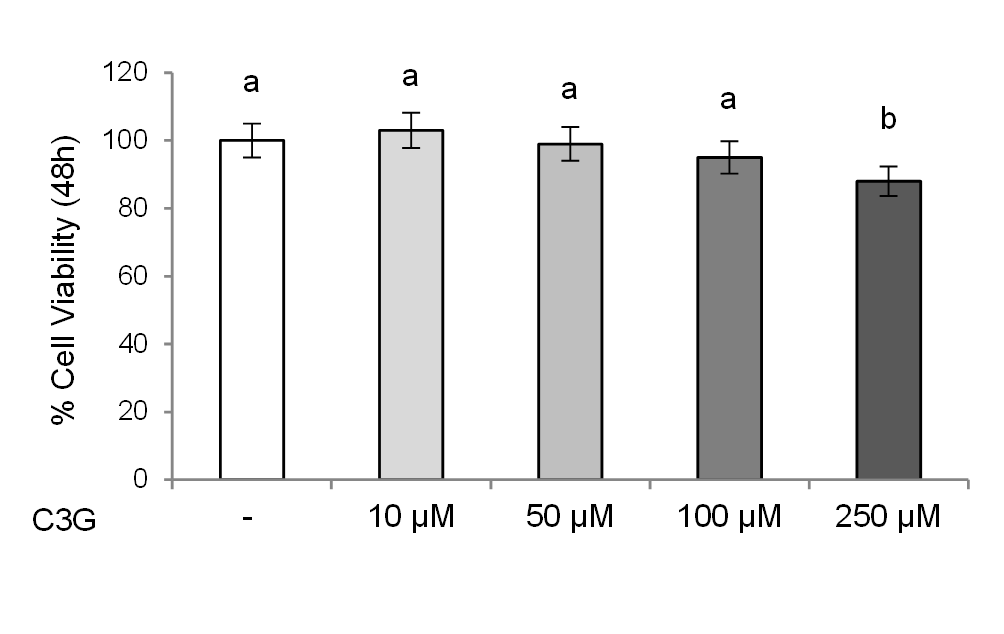


**Supplementary Figure 1.** Cell viability. Cytotoxicity was evaluated by sulforhodamine B assay on 3T3-L1 preadipocytes exposed to different concentrations (10-250 µM) of C3G for 48 h. Control cells were treated with the vehicle (DMSO) alone. Results are expressed as mean ± S.D. of four independent experiments. Means with the same letter are not significantly different from each other (p > 0.05).
